# Deep learning-based analysis of macaque corneal sub-basal nerve fibers in confocal microscopy images

**DOI:** 10.1186/s40662-020-00192-5

**Published:** 2020-05-08

**Authors:** Jonathan D. Oakley, Daniel B. Russakoff, Megan E. McCarron, Rachel L. Weinberg, Jessica M. Izzi, Stuti L. Misra, Charles N. McGhee, Joseph L. Mankowski

**Affiliations:** 1 Voxeleron LLC, San Francisco, CA USA; 2 Department of Molecular and Comparative Pathobiology, Johns Hopkins University School of Medicine, Baltimore, MD USA; 3 Department of Ophthalmology, Faculty of Medical and Health Sciences, New Zealand National Eye Centre, University of Auckland, Auckland, New Zealand

**Keywords:** Cornea, Sensory nerves, Macaque, Deep learning, In vivo confocal microscopy

## Abstract

**Background:**

To develop and validate a deep learning-based approach to the fully-automated analysis of macaque corneal sub-basal nerves using in vivo confocal microscopy (IVCM).

**Methods:**

IVCM was used to collect 108 images from 35 macaques. 58 of the images from 22 macaques were used to evaluate different deep convolutional neural network (CNN) architectures for the automatic analysis of sub-basal nerves relative to manual tracings. The remaining images were used to independently assess correlations and inter-observer performance relative to three readers.

**Results:**

Correlation scores using the coefficient of determination between readers and the best CNN averaged 0.80. For inter-observer comparison, inter-correlation coefficients (ICCs) between the three expert readers and the automated approach were 0.75, 0.85 and 0.92. The ICC between all four observers was 0.84, the same as the average between the CNN and individual readers.

**Conclusions:**

Deep learning-based segmentation of sub-basal nerves in IVCM images shows high to very high correlation to manual segmentations in macaque data and is indistinguishable across readers. As quantitative measurements of corneal sub-basal nerves are important biomarkers for disease screening and management, the reported work offers utility to a variety of research and clinical studies using IVCM.

## Background

In vivo confocal microscopy (IVCM) of the cornea allows for non-invasive acquisition of two-dimensional images, enabling detailed corneal sensory nerve fiber assessment in both clinical and research settings. As the most innervated tissue in the human body, the cornea offers insight into sensory neuropathy rendering the clinical applications of its imaging widespread. Quantitative measurements of corneal sub-basal nerves are, therefore, important biomarkers for disease screening and management. Measures of corneal sub-basal plexus nerve fiber count, density and length have been reported as having clinical utility in diabetes [1, 2], human immunodeficiency virus [3], Parkinson’s disease [4], multiple sclerosis [5, 6], as well as a number of other systemic illnesses. Animal models of these and other diseases play an important role in understanding the disease processes as efforts toward developing new and effective therapeutics are made. The manual derivation of these metrics, however, is time consuming, requires expertise, and is inherently subjective. Automation is therefore necessary and will facilitate standardized analyses across centers as researchers investigate new endpoints in wide ranging clinical applications. As noted by Dabbah [7], this lack of standardized assessment of corneal sub-basal nerve fiber density is a major limitation to wider adoption in clinical settings. Furthermore, the lack of a commonly accepted robust automated analysis method that provides centralized processing limits large-scale multicenter trials.

Several different approaches have been used to automate the task of nerve fiber tracing in IVCM. The challenging image conditions of noise, intensity heterogeneity and low contrast features are compounded by the presence of dendritic, epithelial and inflammatory cells that can have similar features to the nerves being delineated. Parissi adopted a graph-traversal method that traces between seed-points, which is an excellent way to describe the path of a nerve as the method effects constraints on feasible deviations of the nerve’s path and also bridges regions where the nerve’s intensity diminishes given the confocal nature of the modality [8]. Fundamental to the success of such an approach is the choice of start and end points of the graph as to work these must belong to the same nerve. The original method for seed point detection was described by Scarpa, where the image is covered in a grid of evenly spaced line-rows and columns [9]. Nerves are detected at the intersection of lines based on intensity, and a tracing approach is used to follow the nerve in a direction perpendicular to its highest gradient. A final classification uses fuzzy c-means. Dabbah put an emphasis on carefully constructed filter banks applied as a feature detector and enhancing the nerves in accordance to their localized and dominant direction [7]. A final binary image of nerves is created using a global threshold and skeletonization of the result. This work has resulted in the freely available ACCMetrics tool, widely accepted as a standard in clinical IVCM image analysis [2, 7, 10–13].

More advanced machine learning techniques have been added to such processing pipelines for a more sophisticated final arbitration between nerve fiber and background. Guimarães added a pixel-by-pixel classifier to hysteresis thresholded images to create a binarized nerve map [14]. The features that fed the classifier were intensity based and included edge magnitudes. Structure enhancement used log-Gabor filters, and the method facilitates fast processing of large datasets. Annunziata developed a curvilinear structure model employing a set of filter banks to perform feature detection in the image [15]. Additionally, contextual information was added via learned filters and a final classification then takes both of these results to yield an estimate at each pixel of nerve and background. The approach is reliant on both manual tuning and supervised learning for the feature designs, parameters and finally the thresholding. The results using cross validation (CV) are impressive.

It can be seen that, in general, these methods have evolved around carefully designed filter banks acting as feature extractors with a final classification step. The best example of this is the ACCMetrics tool, which, being developed using clinical data, offers a solution to the analysis of human corneal nerves acquired within a clinical environment. The methods, as implemented, should not, therefore, be expected to work “out of the box” on macaque data. As macaque models are used in a variety of diseases characterized by corneal sensory nerve fiber loss, we have developed and characterized a novel approach for automated analysis of nerve fibers, leveraging more current technologies in the world of computer vision and machine learning to process macaque IVCM images that are inherently of lower quality than human ICVM image [16–20].

Recent advances and superior levels of performance seen in the use of deep convolutional neural networks (CNNs) has resulted in their widespread adoption for a variety of image recognition tasks [21, 22]. The deep learning paradigm is to learn both the feature extraction (filters) and classifier using CNNs and supervised learning. The CNNs are capable of building rich, layered (deep) representations of the data which are then classified through additional layers of representation and learned associations. Such significant technical advances in supervised learning have already been successfully applied to automatically tracing corneal nerves in IVCM using clinical data [23, 24]. This study reports on using similar deep learning-based architectures for the automated tracing of corneal nerve fibers in IVCM images of macaque corneas.

## Methods

### Data

All data reported in this study are from archived IVCM images acquired from anesthetized pigtailed macaques (*Macaca nemestrina*) using the Heidelberg HRTIII outfitted with the Rostock corneal module; the macaque image acquisition protocol was approved by the Johns Hopkins University IACUC and all animal work was in accordance with the guidelines outlined in the Animal Welfare Act and Regulations (United States Department of Agriculture) and the 8th edition of the Guide for the Care and Use of Laboratory Animals (National Institutes of Health).

A single scan acquired images at different depths at up to 30 frames a second. In all cases, each image covered a field of view of 400 × 400 μm over 384 × 384 pixels. Using this information, the total lengths (mm) of the tracings can be converted to a measure of nerve length per image (mm/mm^2^). This follows the convention of Dabbah in reporting the corneal nerve fiber length (CNFL), defined as the sum of the length of all nerves per image [12]. For ground-truthing, sub-basal nerves were traced by experienced readers using the ImageJ plugin, NeuronJ [25]. Importantly, this is done at the pixel level; that is, the results of the labelling are images with each pixel labeled as belonging to either nerve or background.

In total, 104 sub-basal plexus images were acquired from 35 macaques. Each was manually selected from all acquired images based on quality and accurate positioning at the sub-basal plexus. The data was split into two parts to (1) assess different CNN architectures using CV and (2) validate the best performing model in an inter-observer study. The split used was 58 images from 22 macaques, and the sequestered data comprised 46 images from 13 macaques.

#### Automated nerve Fiber assessment

Based on the manually labelled data, a supervised deep learning approach to semantic segmentation was used to associate input images to ground truth. This is done by presenting the network, the image data, and labels to create pixel-wise associations. Categorical cross-entropy was used as the loss function that is minimized using backpropagation. The output of a trained network is a nerve probability map where pixels are in the range 0, indicating no nerve data, to 1, just nerve data.

### Pre-processing and post-processing

**Fig. 1:**
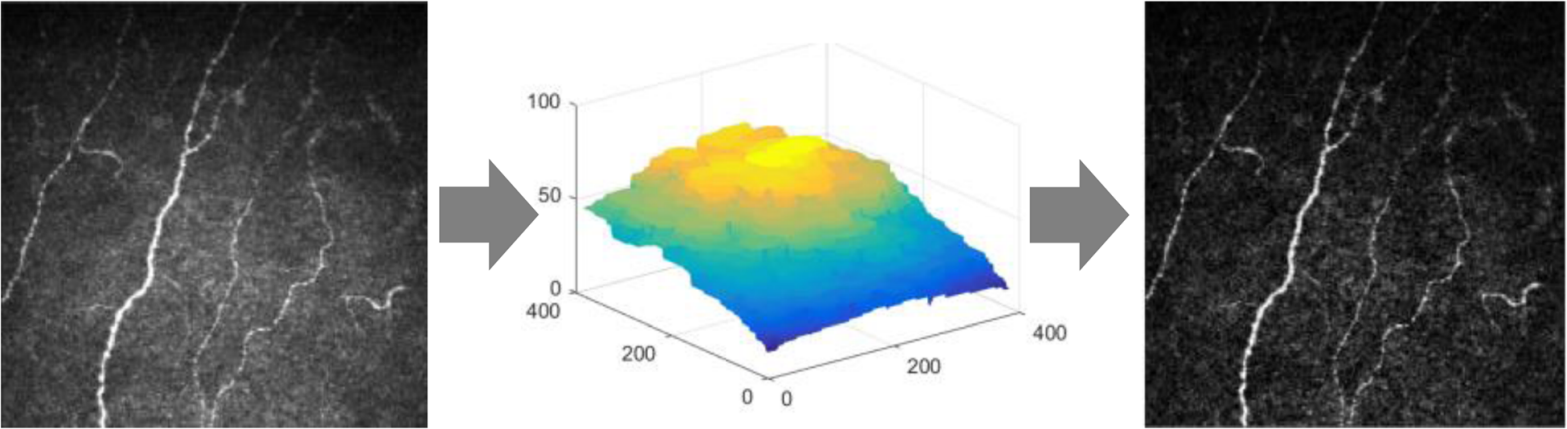
Pre-processing of the input images using background subtraction. The input images are first pre-processed using a simple flat-fielding technique. On the left is an example input image. Its background intensity is estimated using morphological opening (center image). These background intensities are then subtracted from the original image to produce the image on the right showing more even contrast across its entire field of view

Prior to presentation to the network, a pre-processing step is used to account for differing background illumination across the image (Fig. 1). This effect is all the more pronounced in macaque images given the increased curvature of the cornea in the macaques resulting in a faster roll-off of image intensities toward the periphery of the image than seen in human clinical data. Background illumination is first estimated by opening the image using grayscale morphology and a large structuring element, which is a circle with a radius of 4 μm (10 pixels). It then subtracts that result from the original image to reduce the effect of changing background intensity. This is known as top hat filtering, where in this case, the brighter structures smaller than the structuring element are preserved.

Post-processing is applied to the network’s output to threshold the probabilities, in the range 0 to 1, and return a final, binary result. Hysteresis thresholding with a lower value of 0.125 and an upper value of 0.275 are used for all cases in this study. The binary image is then skeletonized [26] and components of less than 35 pixels are removed from consideration.

**Fig. 2:**
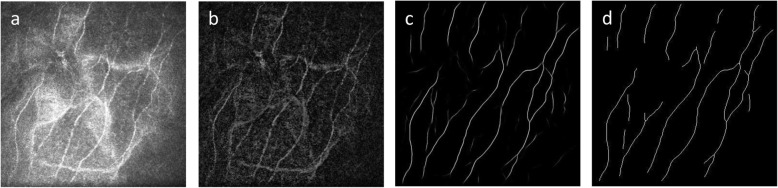
The entire processing pipeline used in this study. The input image (**a**) is pre-processed to compensate for variation in the background illumination (**b**). The segmentation, performed using deep learning, generates a probability image that assigns a score between 0 and 1 to each pixel (**c**) depicting a pixel-wise nerve classification. The final post-processing step is to binarize and skeletonize that result (**d**)

The combination of illumination correction on the front end and hysteresis thresholding on the back end works well in this application where the confocal nature of the imaging system means that nerve fibers can come in and out of view (focus); that is, one must be locally sensitive and adaptive to the imaging conditions. The overall processing pipeline is illustrated in Fig. 2, with the free parameters of the method given above; in the case of the flat-fielding, it is simply the size of the morphological structuring element used to create the background image, and for post-processing it is the thresholds and the minimum individual component size. While the training stage can take hours, the final analysis takes, on average, with the image data in memory, 0.3 s per image on an i7 PC using an Nvidia GTX 1080 graphics card for pre-processing, inference and post-processing.

### Neural network architectures

Three similar architectures for semantic segmentation were assessed, where candidate architectures were limited to networks that learned image to image mappings as a per-pixel classification. Common to the three architectures studied are encoding and decoding processing paths used to generate a final segmentation. Experiments involved altering the depth of the networks and also the optimization parameters, such as learning rate, decay and choice of optimization algorithm as discussed in more detail below.

The first architecture is the autoencoder network [27], as previously reported [28]. The second, a U-Net [29], is basically an autoencoder network with additional connections across the encoding and decoding paths. The third is an extension of this method that adds skip connections within the encoding and decoding paths. This latter approach does this using residual branches that, in alleviating the vanishing gradient problem, facilitates deeper networks to be trained [30]. Performance is gauged using the coefficient of determination (R^2^) to assess strength of correlation between manual and automated results, as well as Bland-Altman analysis.

### Deep learning and cross validation (CV)

The different deep learning architectures were evaluated using 5-fold CV, a standard approach for splitting train and test steps. The aforementioned pre- and post-processing steps were fixed for each as these admit to minimal parameterization as previously reported [31]. While these parameters could also be learned using the CV paradigm, they were simple enough to tune manually once and left alone for all further experiments.

Importantly, in application of CV, folds were chosen such that a single subject did not appear in both the training and test sets. For each fold, the training sets were additionally randomly split at each epoch into 90% training and 10% validation thereby allowing us to gauge how well the model’s learning was proceeding and when it should stop. This common practice is key in being able to decide on the optimizer used, batch sizes, as well as other hyper-parameters such as learning rate and learning decay as, in general, the loss value should decrease in a progressive way as the network learns.

Results for all subjects and folds were pooled and compared to results using other architectures and hyper-parameters. For each of these experiments, a final correlation score between lengths reported by the manual tracings and those from the automated approach allowed us to rank the performance of the different implementations and derive the best model for each of the architectures used.

## Results

### Cross validation data and results

**Fig. 3:**
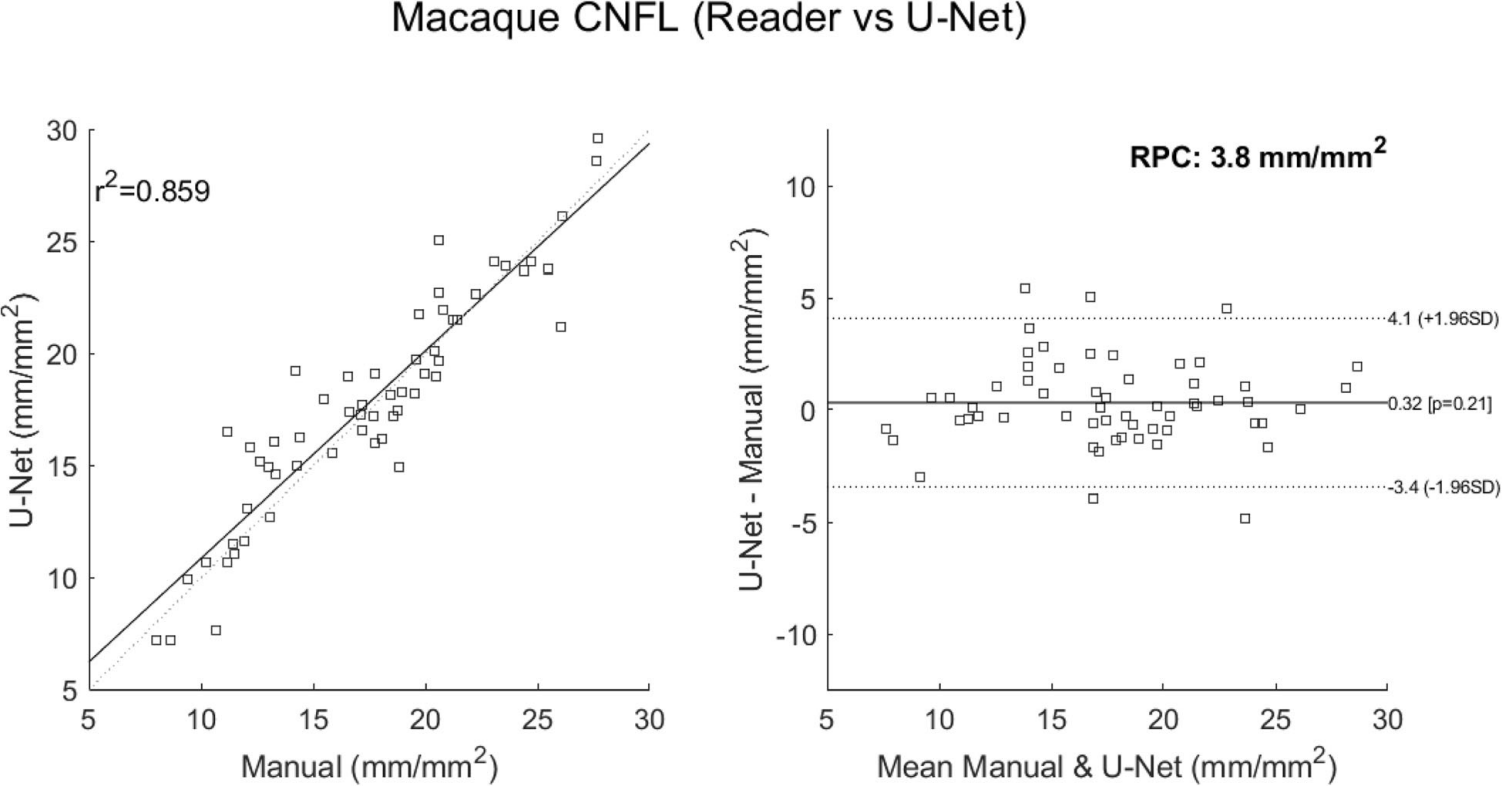
U-Net versus manual tracing correlation and Bland-Altman plots (CV data). Correlations for the best performing U-Net result – deepNerve – to the manual result for the macaque data using 5-fold CV (left). The limits of agreement show no systematic differences as the manual count increases or decreases. The reproducibility coefficient (RPC), 1.96 × SD, is 3.8 mm/mm^2^ (mean difference is 0.3 mm/mm^2^)

**Fig. 4:**
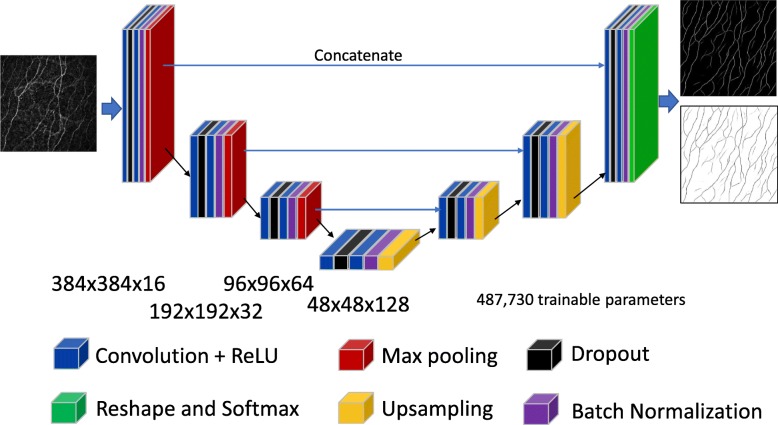
The best performing U-Net architecture used in these analyses, titled deepNerve. Depiction of the U-Net architecture showing both the encoding and decoding paths and their dimensions. For the given input image (left), it outputs two images giving the probability score, at each pixel, of belonging to one of the two classes (right). The final softmax layer ensures these are normalized and can thus be interpreted as probabilities

This dataset comprised 58 IVCM images taken from 22 different macaques. To embellish the data, significant augmentation involving random rotations, skews and flips of the images was used during the training process. Summary correlations between the manual tracing and the automated approaches are given in Table 1 below, where we have included the result using ACCMetrics, a clinical tool applied here to macaque data. Overall, the best performing architecture was the U-Net (Fig. 3). Its configuration is given in Fig. 4, where the optimizer used is the ADAM [32] over 650 epochs; with the learning rate initialized at 10^− 3^, dropping 5^− 4^ every 10 epochs; a batch size of 8; and a drop-out rate of 0.2. In the tradition of naming models for given applications, we refer to this configuration as deepNerve for the remainder of this paper.

**Table 1:** Cross validation performance for the macaque data (*N* = 58 from 22 subjects)

Architecture	5-Fold CV R^2^	Number of trainable parameters
**Autoencoder**	0.733	1,330,498
**U-Net (deepNerve)**	0.859	487,730
**U-Net with Residual Branches**	0.731	516,578
**ACCMetrics**	0.718	N/A

*CV=* cross validation

R^2^: coefficient of determination

In this paper, we interpret the correlation coefficients using Hinkle’s criteria [33] of: very high (≥0.9), high (≥0.7 and < 0.9) and moderate (≥0.5 and < 0.7).

### Inter-observer analysis method

**Fig. 5:**
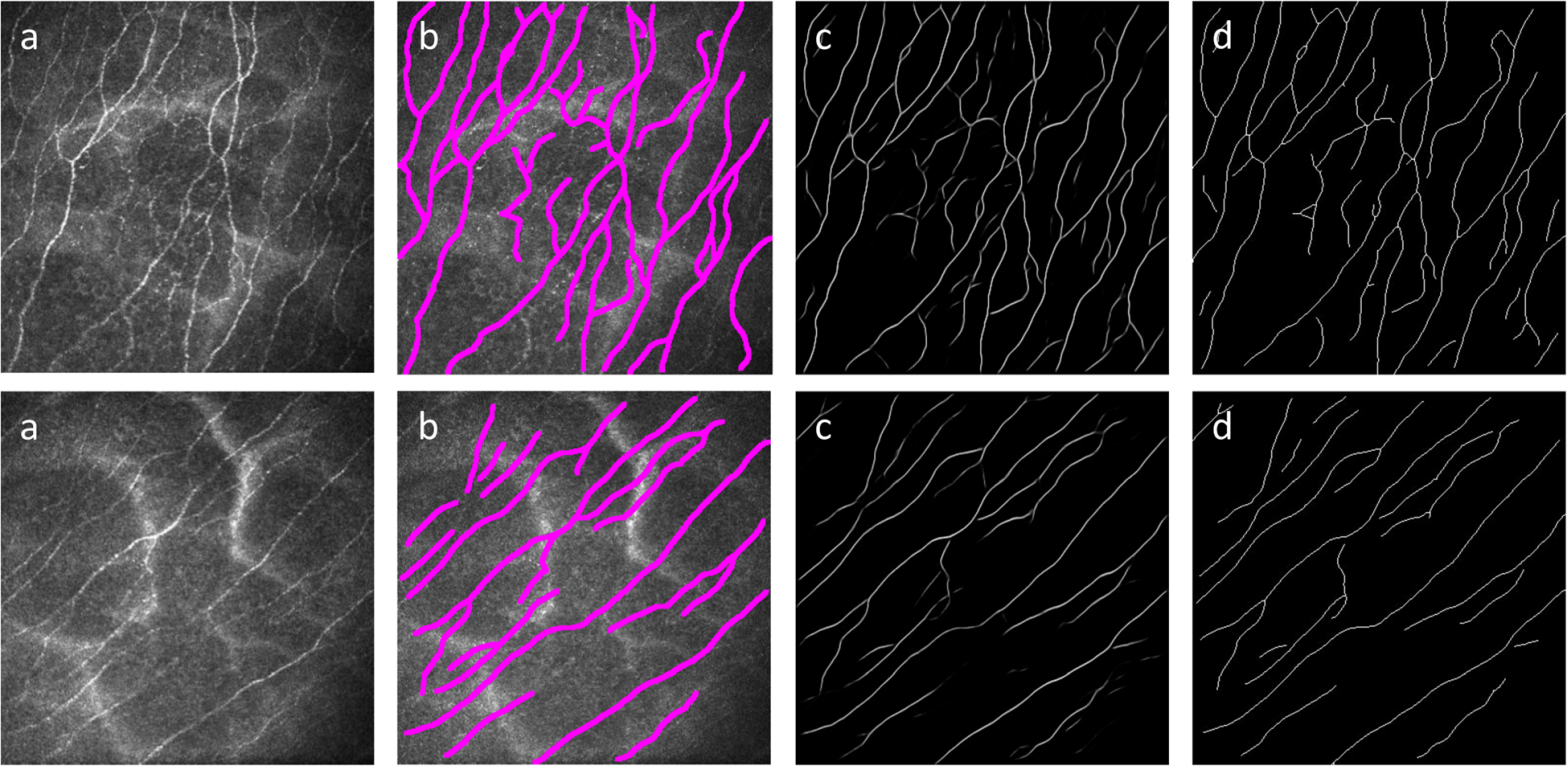
Example macaque data used during the inter-observer evaluation study. The images in the first column (**a**) are input images; the second column shows their manual tracings (**b**); the third column images are the probability images from the output of the neural network (**c**); and the final column gives those images thresholded and skeletonized (**d**)

The best performing architecture, deepNerve of Fig. 4, was then trained on all folds and then frozen for inter-observer analysis; i.e., using all 58 images from 22 macaques in the CV data set. We then applied this model to the sequestered group of 46 images from 13 macaques to validate performance. In this case, three readers were used to independently trace all images (*N* = 46). This allows us to first see how well the best performing model can generalize to truly unseen data, and also to understand how well, in such an applied environment, it performs with respect to expert readers. Example manual and automated segmentation results from this part of the study are given in Fig. 5.

Coefficients of correlation and intraclass correlation coefficients (ICCs) from two-way ANOVA analysis were derived to compare all readers and deepNerve. The results are shown in Table 2 with scores ranging from borderline high (≥0.7) to very high (≥0.9) [33]. The higher the value, the more the agreement, overall, between the two readers. Comparing average correlation scores for each reader relative to all other readers including deepNerve, yields R^2^ values of 0.79 (R1), 0.71 (R2), 0.81 (R3) and 0.80 (deepNerve). The ICC score across all four observers was 0.84, the same as the average ICC score between deepNerve and all individual readers. Of note, the manual tracings that were used to train the network on the previously acquired data were from reader 1.

**Table 2:** R^2^ correlations and intraclass correlation coefficients across all readers

Reader	R1		R2		R3	
Metric	R^2^	ICC	R^2^	ICC	R^2^	ICC
**R2**	0.69	0.84				
**R3**	0.82	0.87	0.75	0.80		
**deepNerve**	0.85	0.85	0.69	0.75	0.85	0.92

*ICC=* inter-correlation coefficient

R^2^: coefficient of determination

### Comparison to the average reader

While CNFL is the biomarker reported most commonly in the literature, it is invariant to location, and the concordance of this value relative to one that is manually derived is not necessarily a good indicator of accuracy of tracings. To assess this and gain confidence in our automated reporting, we used the Jaccard Index (JI), or intersection-over-union, to report the overlap of the automated versus the manual segmentation; a value of 1 being perfect overlap. Given each traced nerve is only one-pixel thick, we define the intersection to be within three pixels to account for insignificant differences. Average and standard deviation JI scores are given in Table 3 below.

**Table 3:** Average and standard deviation Jaccard Indices across readers

Reader	R1	R2	R3
**JI Average**	0.87	0.80	0.85
**JI STD**	0.04	0.08	0.06

*JI=* Jaccard index

*STD=* standard deviation

**Fig. 6:**
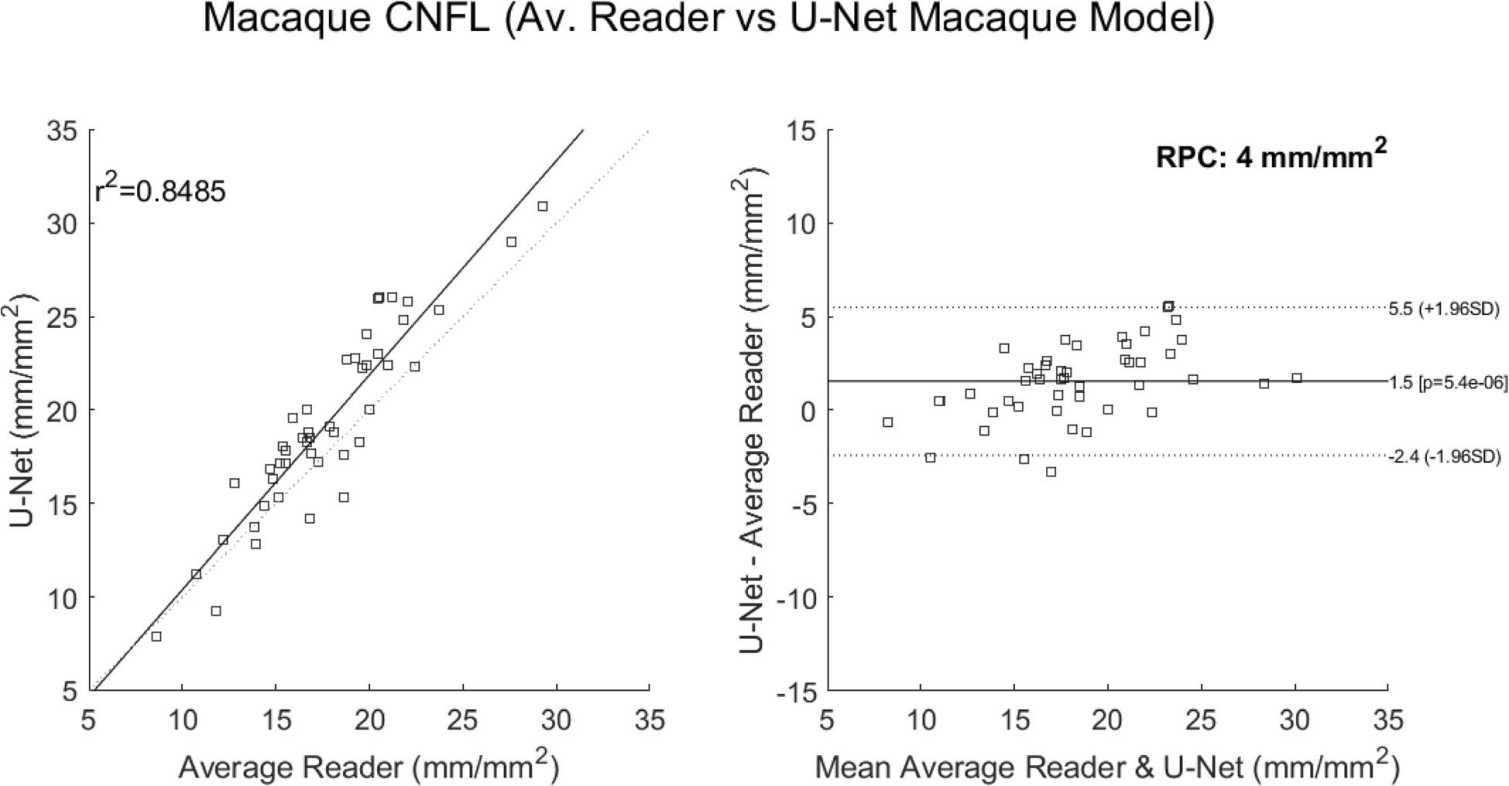
U-Net versus manual tracing correlation and Bland-Altman plots (sequestered data). Application of the best performing U-Net architecture of deepNerve to the sequestered 46 images from 13 new subject macaques. This is our best performing method for the analysis of macaque data. The reproducibility coefficient (RPC), 1.96 × SD, is 4 mm/mm^2^ (mean difference is 1.5 mm/mm^2^)

**Fig. 7:**
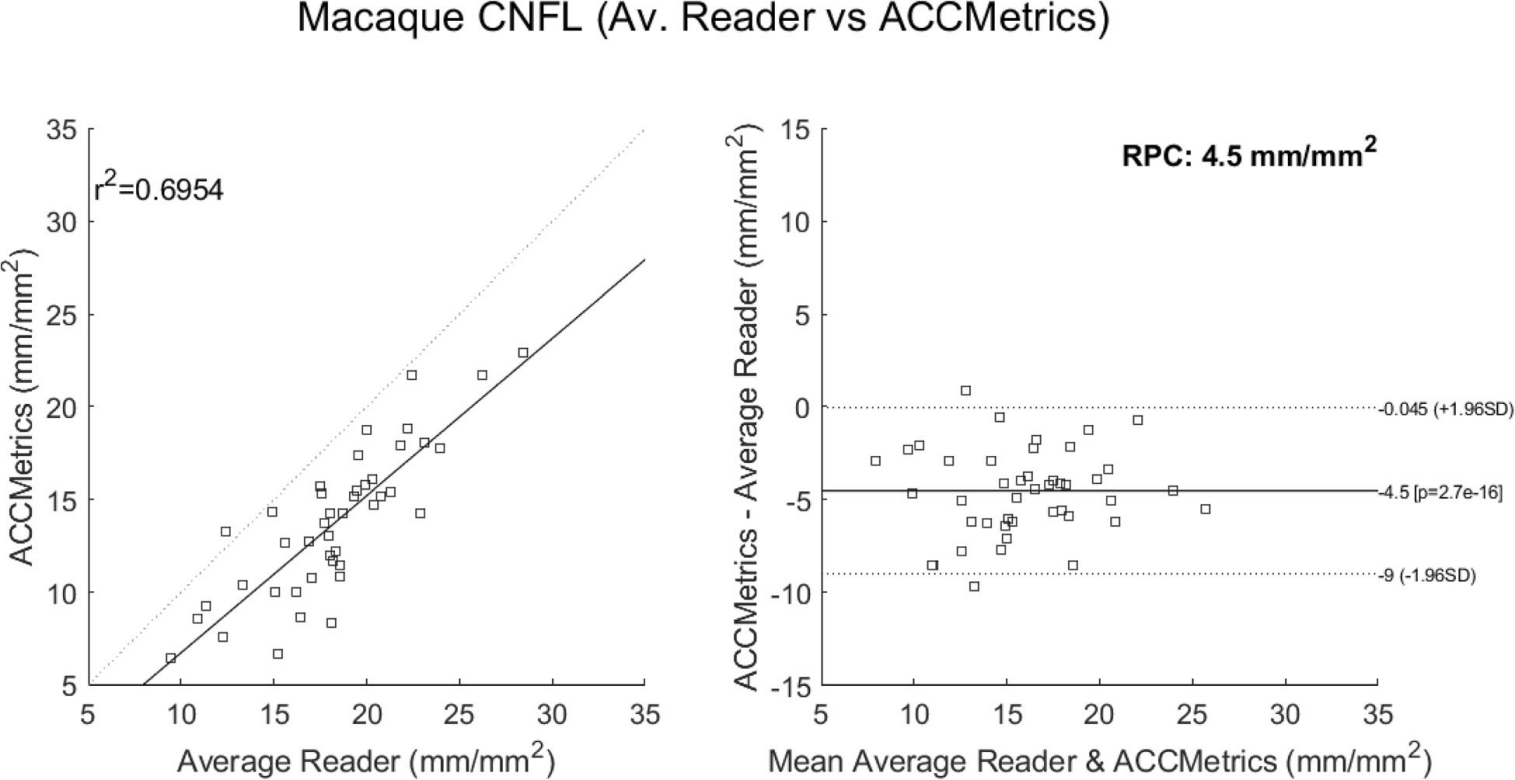
ACCMetrics versus manual tracing correlation and Bland-Altman plots (sequestered data). Application of ACCMetrics to 43 of the 46 macaque images (three images did not process). Note that ACCMetrics was developed using clinical data, so the performance is expected to degrade using this data set. The reproducibility coefficient (RPC), 1.96 × SD, is 4.5 mm/mm^2^ (mean difference is − 4.5 mm/mm^2^)

**Fig. 8:**
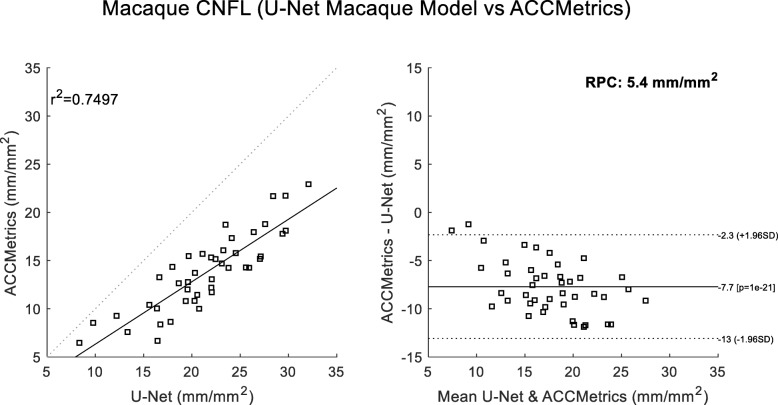
ACCMetrics versus U-Net correlation and Bland-Altman plots (sequestered data). Comparison of deepNerve to ACCMetrics for the CNFL parameter (*N* = 43) shows that the U-Net in general reports higher values. The reproducibility coefficient (RPC), 1.96 × SD, is 4.5 mm/mm^2^ (mean difference is − 5.4 mm/mm^2^)

**Fig. 9:**
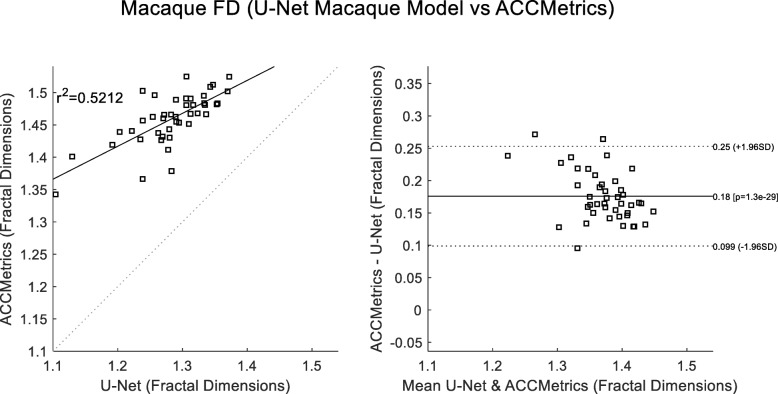
Fractal dimension scores of ACCMetrics versus U-Net correlation and Bland-Altman plots (sequestered data). Comparison of the reported Fractal Dimensions of deepNerve and ACCMetrics (N = 43) shows that the measurements are moderately correlated. The reproducibility coefficient (RPC), 1.96 × SD, is 0.077 (mean difference is 0.18)

A final comparison looked at correlations to the average reader CNFL values. These considered 1) deepNerve (Fig. 6) and 2) ACCMetrics applied to the sequestered macaque data of 46 images (Fig. 7). For deepNerve, the limits of agreement (LOA) about the mean are given by the reproducibility coefficient (RPC) of 4 mm/mm^2^. For ACCMetrics, the RPC increased to 4.5 mm/mm^2^. For both cases, the LOA show no apparent bias, and the correlations are high [33], with deepNerve being at 0.85 and ACCMetrics at 0.70. The means (and standard deviations) of the CNFL parameter for the average reader, deepNerve and ACCMetrics are: 18.28 (3.92), 21.46 (5.34), 13.75 (3.99), respectively. A direct comparison of CNFL between deepNerve and ACCMetrics is given in Fig. 8, and we also looked at the derived parameter, fractal density, for deepNerve and ACCMetrics, as reported in Fig. 9.

There is widespread interest in using in vivo basal-nerve density assessment as a biomarker gauging corneal sensory nerve fiber loss. This is because of its relevance in a number of neuropathies and also systemic diseases. With such interest comes the need to automate the analysis, which, ahead of clinical adoption, also requires a validation of the approach. This study reports on a means of automating the analysis, here leveraging state of the art segmentation methods based on deep CNNs. It also presents a validation of this approach as applicable for use with macaque data. Furthermore, given a robust segmentation, derivative measures such as fractal density [34] and tortuosity [15] may also be added to the armamentarium of biomarkers supportive of this modality.

The approach documented herein builds upon our original methods that were applied originally to ex vivo studies of immunostained corneal whole mounts. Its extension to in vivo data and corneal confocal imaging using deep learning was motivated by our preclinical research using animal models. Such research is important to understanding disease mechanisms as well as response to therapeutics; and by extension, the analysis method is also clinically relevant, particularly if the reported performance can be achieved with clinically acquired data from human subjects. This would require additional validation studies [2].

Comparison is made in this work to ACCMetrics, but only to reference an alternative method as this software has not been developed for non-human analysis. This is an important caveat as firstly the anatomy is different with a known increase in curvature of the cornea in the macaques and, in our review, macaque nerves may be generally thinner. With that said, the performance is still good, has strong correlation to the manual readers, and no exhibited bias in the Bland-Altman analysis. This serves, therefore, to speak to the overall robustness of the implementation. It also forewarns that, to apply our technique to clinical data, we will likely have to re-train the algorithm using just clinical data, as might be expected. Given the successful application of a similar approach to clinical data, Colonna and Williams have both firmly established this to be a method that can be directly applied to human data [23, 24]. Interestingly, both use the same U-Net architecture, although Williams et al. apply the network to patches of the entire image, taking a majority decision where the patches overlap [24].

The limitations of this study relate firstly to the limited amount of data used. We attempt to address this by [1] utilizing extensive data augmentation together with the well-established technique of cross-validation in comparing the different CNN models’ performance; and [2] by using a sequestered data set for the inter-observer analysis. In both cases, we consider the reported performance to be characteristic of performance in unseen data. A second limitation is a comparison to existing techniques. This is hard to circumvent as this paper presents the first method explicitly developed for use in macaque data. The comparison to ACCMetrics is done as this is a validated clinical approach, so while not strictly applicable, it is the most established technique and one to which all new methods should be contrasted. It should also be noted that, in using a supervised learning approach, we are requiring that the input data be from the correct corneal plexus and do not, in general, contain structure from outside of the sub-basal layer. We currently manually select these images from the entire confocal stack but are also working on methods to automate this.

## Conclusions

In summary, we present a novel approach to the analysis of sub-basal nerves in IVCM imaging of macaque corneas. In conjunction with relatively simple pre- and post-processing, excellent correlation with manual readings was achieved. In a comparison across observers, we see that deepNerve is indistinguishable from manual tracings. Lastly it should be noted that, while IVCM is currently the modality of choice, translation to other modalities should only require retraining of the neural network. There is, for example, increasing interest in the use of optical coherence tomography (OCT), the imaging standard of care in ophthalmology, for corneal nerve imaging [35]. If such a scenario evolves, this would make clinical adoption all the more likely in the future given the proliferation of OCT devices and the ease of acquisition.

## Data Availability

All data generated or analyzed during this study are included in this manuscript.

## Appendix

The U-Net implemented to realize the segmentation used Keras version 2.2.4 and TensorFlow 1.12. The convolution layers shown in Fig. 4 use Conv2D, with the filter sizes as given in the figure. Both dropout and batch normalization are used, ordered as in the figure: dropout occurring between the convolution layers, batch normalization after convolution, but before max pooling. So, for example, a single encoding block would look like:

conv2 = Conv2D(192_sz, (3, 3), activation='relu', padding='same', data_format='channels_last')(pool1)

conv2 = Dropout(drop_out_rate)(conv2)

conv2 = Conv2D(192_sz, (3, 3), activation='relu', padding='same', data_format='channels_last')(conv2)

conv2 = BatchNormalization()(conv2)

pool2 = MaxPooling2D((2, 2))(conv2)

## Abbreviations

CNFL: Corneal nerve fiber length
CNN: Convolutional neural network
CV: Cross validation
IVCM: In vivo confocal microscopy
ICC: Inter-correlation coefficient
JI: Jaccard index
LOA: Limits of agreement
OCT: Optical coherence tomography
